# Increased whole blood FFA2/GPR43 receptor expression is associated with increased 30-day survival in patients with sepsis

**DOI:** 10.1186/s13104-018-3165-4

**Published:** 2018-01-16

**Authors:** Zyad J. Carr, Andry Van De Louw, Graham Fehr, Jialiu D. Li, Allen Kunselman, Victor Ruiz-Velasco

**Affiliations:** 1 Department of Anesthesiology & Perioperative Medicine, Penn State Milton S. Hershey Medical Center, H187, 500 University Dr., Hershey, PA 17033 USA; 2 Division of Pulmonary & Critical Care Medicine, Penn State Milton S. Hershey Medical Center, Hershey, PA 17033 USA; 3 Department of Public Health Sciences, Penn State College of Medicine, Penn State Milton S. Hershey Medical Center, Hershey, PA 17033 USA

**Keywords:** GPR43, FFA2, Sepsis, Short-chain fatty acids, Multiple organ dysfunction

## Abstract

**Objective:**

Sepsis is a condition associated with a dysregulated inflammatory response to infection with significant morbidity. Recent advances have elucidated the vital role that the short chain fatty acid glycoprotein receptor 43 (FFA2/GPR43) plays in inflammatory and immunomodulatory pathways. We hypothesized that elevated whole blood GPR43 RNA expression would be associated with increased 30-day survival in patients admitted with sepsis. Patients (n = 93) admitted to the intensive care unit with the diagnosis of sepsis underwent quantitative real time PCR within 48 h of intensive care unit admission. Clinical and demographical parameters were retrospectively extracted from the chart and compared to quantitative measurements of GPR43 RNA expression.

**Results:**

Utilizing logistic regression, we found that the odds of mortality decreased for every one-unit increase in GPR43 RNA expression for patients that survived to 30 days [OR = 0.71; 95% CI (0.50, 0.99) p = 0.049]. Using linear regression, we determined that the increase in whole blood GPR43 expression was not associated with whole blood white cell count [r = 0.04; 95% CI (−0.16, 0.24); p = 0.70] or body mass index [r = − 0.07; 95% CI (− 0.23, 0.18); p = 0.81]. We conclude that the GPR43 receptor plays an integral role in survival during and after sepsis.

## Introduction

Sepsis is a condition associated with a dysregulated inflammatory response to infection and despite advancements in early recognition and treatment, continues to be a frequently fatal condition. It is a common reason for hospital admission in the United States, comprising 750,000 cases with an estimated death rate of 28–38% [1]. Predictably, patients with shock associated with sepsis have the highest mortality rate of approximately 50% [2]. The pathways involved in the progression from sepsis to septic shock and multiple organ dysfunction syndrome (MODS) remains partly unknown. Dysfunction at the level of the gut may be associated with abnormal translocation of bacteria, dysbiosis of the intestinal microbiome or release of intestine derived mediators that may cause distal organ injury [3–7].

G protein-coupled receptors (GPCRs) comprise the largest cohort of transmembrane proteins in the mammalian genome and are involved in a diverse variety of modulatory effects across organ systems. A subfamily which include FFA1 (GPR40), FFA2 (GPR43) and FFA3 (GPR41) have been recently characterized that utilize short chain free fatty acids (SCFA) as ligands [8]. These free fatty acid receptors demonstrate wide differences in SCFA specificity, tissue localization and intracellular signaling. Under normal conditions, GPR43 is highly expressed in immune cells, such as in neutrophils and monocytes, and at higher levels than GPR41 and GPR40 [9]. GPR43 is involved in modulation of intestinal inflammation and neutrophil chemotaxis in mice [10, 11]. In addition, GPR43 plays an essential role in the differentiation of precursor immune cells into key components of host defenses such as monocytes and macrophages, polymorphonuclear (PMN) cell chemotaxis and recruitment to sites of bacterial invasion [12, 13]. Following inflammatory or infectious stimuli, SCFA activate GPR43 receptors on intestinal epithelial cells. This is followed by upregulation of chemokines and cytokines, as part of the coordinated immune response to bacterial infection [14]. Phagocytic chemotaxis requires GPR43 activation to maintain intestinal integrity and immune defenses and GPR43 knockout mice show higher mortality compared to wild type mice in an acute colitis model and reduced intestinal inflammatory changes in a chronic colitis model [15, 16]. To our knowledge, the only human study that has examined GPR43 expression found that fetal placental GPR43 expression was significantly higher in parturients with clinical signs of infection [17]. Based on these previous data, we hypothesized that higher GPR43 RNA expression would be associated with improved survival in patients with the diagnosis of sepsis. We performed a retrospective study analyzing previously obtained whole blood RNA sequencing data in patients with sepsis to determine the relationship between GPR43 RNA expression and the severity of critical illness as delineated by the Sequential Organ Failure Assessment (SOFA) score [18] as well as 30-day and 1-year mortality.

## Main text

### Materials and methods

The methods used in this retrospective data analysis were presented and approved by the Penn State College of Medicine Institutional Review Board and Human Subjects Protection Office (IRB). We examined previously obtained whole blood RNA sequencing data obtained from the IRB approved Penn State Hershey Medical Center Critical Illness Registry. After consent and inclusion in the registry, blood was obtained from patients within 48 h of intensive care unit (ICU) admission. Patients included in this cohort met clinical criteria of age greater than 18 years, and the diagnosis of sepsis or septic shock as defined by: evidence of life-threatening organ dysfunction, suspected or documented infection by a physician, and an acute increase of > 2 SOFA points (Table 1). Patients meeting the definition of septic shock demonstrated the above criteria for sepsis and vasopressor therapy and lactate > 2 mmol/L (18 mg/dL) [19]. SOFA scores were assessed for all eligible patients utilizing clinical variables retrieved from data collected within + or − 12 h of documented ICU admission time. Through retrospective chart review, data including age, gender, body mass index (BMI), white blood cell count (WBC), and suspected source of infection based on admitting history and physical examination were retrieved. Microbiological culture data was retrieved from patients up to 7 days from admission to the ICU. Samples were processed per Department of Microbiology guidelines for retrieval, handling, incubation and monitoring of aerobic and anaerobic microbial cultures. A positive culture was tabulated if there was identification of positive microbial growth of a single species of bacteria in blood, urine or tracheal aspirate samples drawn within 24 h of admission. A polymicrobial infection was tabulated if the samples had two or greater species and was not expressly noted to be normal flora by the final laboratory result.

**Table 1:** The Sequential Organ Failure Assessment (SOFA) score

Variable	SOFA score				
	0	1	2	3	4
Respiratory					
Pa02/Fi02 mmHg	> 400	< 400	< 300	< 200	< 100^a^
Coagulation					
Platelets × 10^3^/μL	> 150	< 150	< 100	< 50	< 20
Liver					
Bilirubin, mg/dL	< 1.2	1.2–1.9	2.0–5.9	6.0–11.9	> 12.0
Cardiovascular					
Hypotension	No hypotension	Mean arterial pressure < 70	Dop < 5 or Dob any dose^b^	Dop > 5, Epi < 0.1 or Norepi < 0.1^b^	Dop > 15, Epi > 0.1, Norepi > 0.1^b^
Central nervous system					
Glasgow coma scale	15	13–14	10–12	6–9	< 6
Renal					
Creatinine, mg/dL or urine output, mL/day	< 1.2	1.2–1.9	2.0–3.4	3.5–4.9 or < 500	> 5.0 or < 200

*Norepi* indicates norepinephrine, *Dob* dobutamine, *Dop* dopamine, *Epi* epinephrine, *Fi02* fraction of inspired oxygen

^a^With respiratory support

^b^Adrenergic agents administered for at least 1 h (doses in µg/kg/min)

### Genomic analysis

#### RNA-Seq library construction and sequencing

RNA was extracted from blood samples using the QIAsymptony PAXgene Blood RNA kit obtained from Qiagen Sciences, Inc (Germantown, MD). We used approximately 200 nanograms (ng) of RNA from each sample to generate RNA-Seq cDNA libraries for sequencing using the Aligent Technologies SureSelect Strand Specific RNA Library Preparation Kit (Santa Clara, CA). Sample preparation followed the manufacturer’s protocol that included the isolation of poly-adenylated RNA molecules using poly-T oligo-attached magnetic beads, chemical RNA fragmentation, cDNA synthesis, ligation of bar-coded adapters, and PCR amplification. The amplified cDNA fragments were analyzed using the 2100 Bioanalyzer from Agilent Technologies, Inc. (Santa Clara, CA) to determine fragment quality and size. cDNA library concentrations were determined by using the Kappa library quantification kit (Kappa Biosystems, Wilmington, MA). Finally, sequencing of 75 base pair single-end reads was performed with an Illumina HiSeq 2500 instrument at the Penn State Hershey College of Medicine Genome Sciences Facility (Hershey, PA).

#### RNA-Seq preprocessing

We imported the raw fastq files obtained from sequencing to the Illumina BaseSpace cloud computing environment (https://basespace.illumina.com), and used the Tophat package to align samples to the UCSC hg19 reference genome [20]. We downloaded a total of n = 128 RNAseq bam files from Illumina BaseSpace, and used samtools to convert them to sam files [21, 22]. We next used HTSeq to compute gene-level read counts based on the hg19 reference genome [23]. We identified and removed genes with low read counts, defined as less than five reads in at least 50% of the samples. This yielded read count data for 12,073 genes. Quality controls data was available for n = 114 samples, and subsequent manual review of read alignment percentages and RNA integrity numbers identified 101 samples that were suitable for analysis. We used the edgeR R package to quantify gene expression using reads per kilobase of transcript per million mapped reads (RPKM) [24, 25]. We batch corrected the log-scale RPKM measurements with the SVA R package [26].

#### Sample size

We used all available patients enrolled in the critical care registry that met eligibility criteria (n = 93) for this retrospective study. Post hoc analysis of the outcomes of 30 day and 1-year mortality determined, for an alpha of 0.05 and a sample size of 93 patients, the type 2 error is 0.006 and the power is 0.994. For variables assessed with linear regression, (α = 0.05, n = 93), the type 2 error is 0.026 and the power is 0.974.

#### Statistical methods

Descriptive statistics are reported as frequencies and percentages for categorical data and as mean and standard deviation (SD) for continuous data. Two-sample t tests were used to assess sex differences with respect to GPR43 RNA expression and SOFA scores. Pearson’s correlation coefficient (r), with associated 95% confidence interval (CI), was used to assess the strength of the correlation between GPR43 RNA expression and the following variables: SOFA scores, BMI, and white blood cell count on admission to the ICU. Logistic regression was used to assess the association of GPR43 RNA expression with clinical outcomes (i.e., 30-day mortality, 1-year mortality, culture positivity, presence of poly-microbial infection). The effect sizes from the logistic regression models were quantified using the odds ratio (OR) and 95% CIs. All hypothesis tests were two sided and all analyses were performed using SAS statistical software, version 9.4 (SAS Institute Inc., Cary, NC).

### Results

**Fig. 1:**
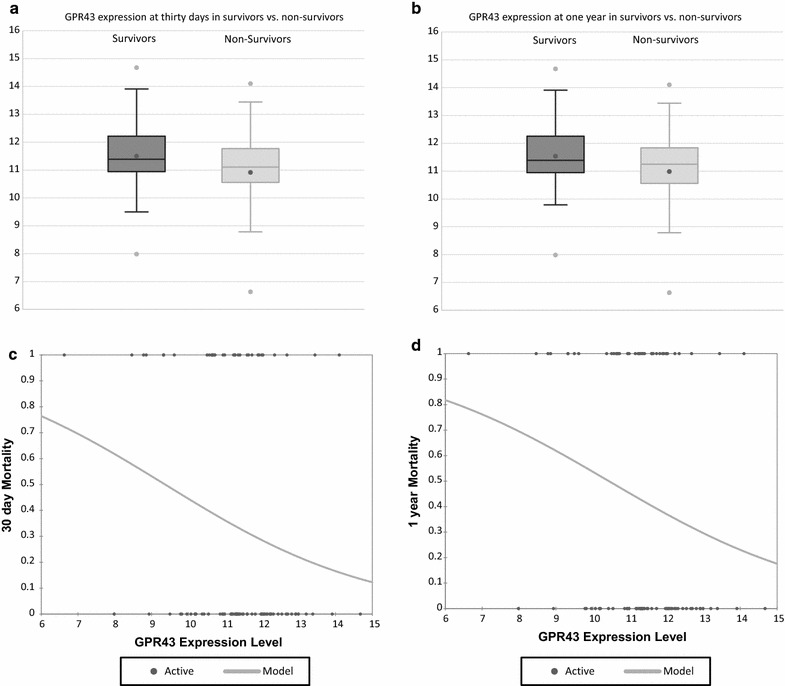
GPR43 expression (log RPKM) is significantly elevated across 30 day and 1 year survivors in comparison to non-survivors (**a**, **b**). The logistic regression plot of the mortality and log RPKM demonstrates consistent though moderate sensitivity to survivorship at both 30 days and 1 year (**c**, **d**)

Demographics of the 93 patients that met inclusion criteria are reported in Table 2. We compared GPR43 expression with several clinical parameters associated with sepsis. There was no evidence of any difference between males and females with respect to either GPR43 RNA expression [r = 0.2; 95% CI (− 0.4, 0.7); p = 0.52] or SOFA scores [r = 0.1; 95% CI (− 1.5, 1.7); p = 0.93]. Secondly, there was no difference of age with GPR43 expression [r = − 0.13; 95% CI (55.94,62.75); p = 0.22] or SOFA score [r = 0.10; 95% CI (8.15,9.74); p = 0.32]. Furthermore, there was no evidence of a correlation between GPR43 RNA expression with SOFA scores [r = − 0.09; 95% CI (− 0.29, 0.11); p = 0.36], BMI [r = − 0.07; 95% CI (− 0.23, 0.18); p = 0.81], or WBC count on admission to the ICU [r = 0.04; 95% CI (− 0.16, 0.24); p = 0.70]. We found no evidence of a relationship between GPR43 RNA expression and the following clinical parameters: positive cultures [OR = 1.02; 95% CI (0.74, 1.39) p = 0.91] or polymicrobial infection [OR = 0.65; 95% CI (0.65, 1.69) p = 0.84]. Evidence was found that the odds of mortality decreased for every one-unit increase in GPR43 expression for both patients that survived to 30 days [OR = 0.71; 95% CI (0.50, 0.99) p = 0.049] and that survived to 1-year [OR = 0.71; 95% CI (0.51, 1.00) p = 0.051] (Fig. 1).

**Table 2:**
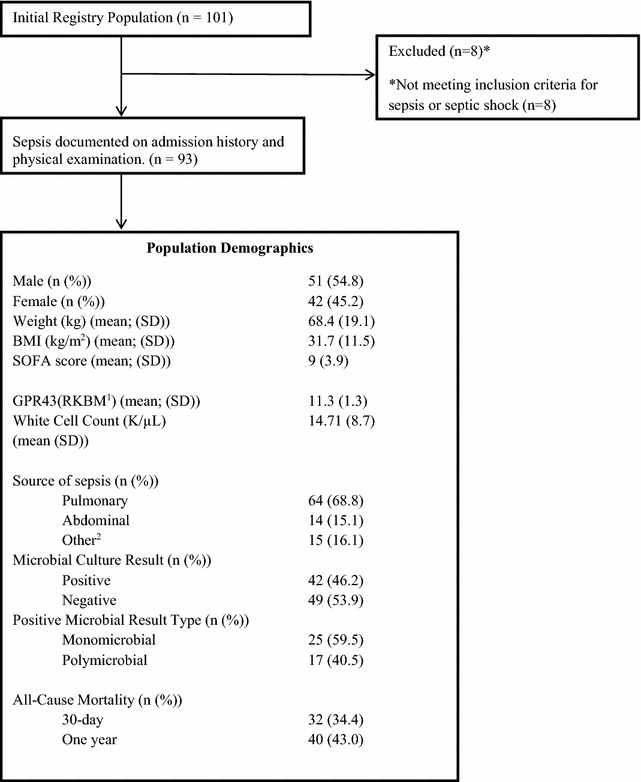
Subject population statistics

^1^Reads per kilobase million (log)

^2^Bloodstream 6.46%, Cardiac 3.23%, Dental 2.15%, Endocrine 1.08%, Neurological 1.08%

### Discussion

In the present study, we have shown evidence that higher GPR43 RNA expression was associated with lower 30-day and neared significance for 1-year mortality in patients with sepsis or septic shock. GPR43 expression was not found to be related to admission WBC count, BMI, gender, or severity of illness nor related to the presence of positive cultures or polymicrobial infections. The lack of correlation to BMI findings were consistent with a study of GPR43 expression in humans which found no difference in obese compared to normal weight subjects [27]. Notably, higher expression of GPR43 was related to improved 30-day survival independent of these factors. GPR43 is highly specific for immune tissues and inflammatory cells and is present disproportionately in neutrophils, monocytes and PMN cells. In an infection model with *C. rodentium* in GPR43 knockout mice, failure to induce the acute inflammatory response on inoculation resulted in failure to clear the infection, delayed induction of inflammatory cytokines and precipitated failure of neutrophil infiltration [14]. Thus, GPR43 mediated PMN migration and activation is likely involved in protective functions at the epithelial level and an inadequate response would lead to a failure to fend off opportunistic intestinal derived pathogens. Polymorphisms have been demonstrated in the human GPR41 gene, and it may play a role in producing a muted immune response in non-survivors [28]. Although it is unclear whether this process occurs via dysfunctional metabolic or immunomodulatory control interactions during the acute phase of sepsis, we believe these findings add to the evidence of the important role played by the gut and microbiome in sepsis.

### Conclusion

To our knowledge, this is the first study that has demonstrated a mortality benefit to increased GPR43 expression in sepsis. In our study, we showed that increased GPR43 expression was related to improved 30-day survival regardless of BMI, sex, WBC count, or presence of positive cultures. The lack of a meaningful relationship between SOFA score and GPR43 expression may be related to the wide variability of MODS, and clinical treatment variation in the subject population. Further research to delineate the specific downstream advantage of increased GPR43 expression in sepsis and its favorable role in immunomodulation are required, preferably with a prospective clinical study. Elucidation of upstream factors that influence GPR43 expression should improve as we refine allosteric GPR43 agonists and antagonists [29]. We conclude that the activity of the GPR43 receptor is integral to survival in sepsis and that further research is warranted to characterize the advantages of increased GPR43 expression.

## Limitations

Our patient population was limited and only 101 patients had gene expression data for analysis. In addition, samples were obtained once within 48 h of ICU admission, and we were unable to assess GPR43 expression throughout the subject population’s entire treatment course. There was a lack of clinical standardization of care within our study population. Lastly, because of the retrospective nature of chart review, confounding factors during data collection cannot be excluded.

## Abbreviations

BMI: body mass index
MODS: multiple organ dysfunction syndrome
GPCRs: Glycoprotein Coupled Receptors
FFA: free fatty acid
GPR: glycoprotein receptor
RNA: ribonucleic acid
IRB: Institutional Review Board
ICU: intensive care unit
SOFA: Sequential Organ Failure Assessment
RNA seq: ribonucleic acid sequencing
PMN: polymononuclear
WBC: white cell count
